# Post craniotomy pain management in Copenhagen rat by intraperitoneal or oral dosage of Tramadol: a comparative evaluation

**DOI:** 10.1038/s41598-023-43330-4

**Published:** 2023-09-26

**Authors:** Sasmita Samal, Debyashreeta Barik, Sarita Jena

**Affiliations:** 1 Institute of Life Sciences, Nalco Square, Bhubaneswar, Odisha 751023 India; 2 School of Biotechnology, Kalinga Institute of Industrial Technology (KIIT) University, Bhubaneswar, Odisha 751024 India

**Keywords:** Fracture repair, Behavioural methods, Animal disease models, Animal behaviour

## Abstract

Calvarial craniotomy in animal models involves pain and distress. Moderate to severe pain in laboratory animals requires adequate pain management strategies. According to previous studies, the options available for suitable analgesia for rat calvarial craniotomy are very few. For most analgesic treatments, injectable routes of administration are predominantly used. However, these routes require restraining the animals, which may cause unnecessary pain, distress and suffering. As a well-fare measure, we focused on pain management by oral administration of analgesia. In this particular study, which is a sub-study of a major experiment on bone regeneration with different polymeric scaffold materials, we have compared the analgesic efficacy of intraperitoneal (I/P) and oral administration of tramadol (10 mg/kg) over a period of 96 h post-surgery in rat craniotomy models. The focus of our study is to evaluate the potential pain reduction efficacy of orally administered Tramadol without any restraining involved. We have used various non-invasive methods to assess the pain-alleviating efficacy of tramadol administered through different methods. We found that the efficacy of oral administration of tramadol is comparable to I/P administration in alleviating pain. Additionally, oral administration through drinking water has the benefit of not putting the animal under unwanted restraining stress.

## Introduction

Bone tissue engineering construct and biomaterial development to assess bone regeneration require a small laboratory animal model before its screening in larger animals and further translation to human applications. The rat model with critical size defect in the calvarial bone is one of the choices for the above validation^1^. The craniotomy model is associated with moderate to severe pain. However, studies involving pharmacological and non-pharmacological pain management of post-craniotomy pain for rat models are very limited. The aim of analgesic management of pain is not only to eliminate the pain but also to achieve a level for the animal to enable basic species-specific activities without compromising their well-being^2^. Null or inadequate pain management can result in physiological and psychological distress. The distress may lead to increased mortality, immune dysfunction, impaired wound healing and affect not only animal welfare but also increase the intra-animal variation leading to altered study outcome^3,4^.

Continuous refinement is required in pain management strategies like the choice of analgesic, dose, route of administration, frequency of administration, duration of the application and the methods of pain assessment. Unfortunately, the choice of suitable analgesic for the rat calvarial bone defect model is very scarce. The selection of analgesics may be a non-steroidal anti-inflammatory drug (NSAID), opioid or opioid analogue. The use of NSAIDs is mostly avoided in osteotomy in pre-and post-operative pain management because of its potential unintended effect on the initial inflammatory phase of bone healing and regeneration^5–8^. Buprenorphine is one of the most common opioid analgesics used in rodents to mitigate post-operative mild to moderate pain. It is a partial µ-opioid receptor agonist and acts for a longer duration without causing respiratory depression and immunosuppression^9–11^. But buprenorphine has certain side effects like sedation, cardiovascular depression, anorexia, and gastrointestinal distress. Furthermore, buprenorphine is mostly administered through subcutaneous (SC) or intraperitoneal (IP) route and administration through oral route are limited due to conflicting opinions on its efficacy^12–14^.

Tramadol is an opioid analogue and one of the potent analgesic agents but supporting data on its efficacy in rats for osteotomy pain management is rare and so also the recommended dosage. Tramadol offers an analgesic effect with both opioid and non-opioid mechanisms of action. It also exhibits very mild adverse effects like respiratory depression and ileus^15^. Tramadol is effective in lower concentrations for acute pain^16^. Some studies suggest that tramadol administration through an injectable route (subcutaneous or intraperitoneal) is effective at a dosage of 12.5–50 mg per kg body weight. However, sedation was the side effect observed at a higher dose and no side effect at lower dose^17^. In our study we have taken a dose of 10 mg/kg^18–21^, as very lower dose of tramadol is not potent to relieve the pain in rats whereas higher dose of tramadol may induce sedation^17^ and increased NSS (numerical seizure score)^19^. It has been seen that, during both at active and resting phase administration of tramadol in drinking water resulted in stable plasma concentration in mice^22^. But we observed that there is no study till now in rats with similar mode of administration. Administration of drugs through non-stressful methods like voluntary intake or self-administration of the drug in drinking water has added benefits to injectable routes by avoiding handling stress.

This sub-study is a part of a major experiment where the effect of implanted biomaterial scaffolds on bone regeneration has been assessed. Pain alleviating efficacy of T^Oral^ (10 mg/kg) or T^I/P^ (10 mg/kg) are comparable. However, T^Oral^ through drinking water has added benefits of refraining the animal to unnecessary distress due to handling and restraining. The pain alleviation was assessed by (a) measurement of feed intake and alteration in body weight, (b) non-invasive methods like rat grimace scale scoring, nest-let integration to nest and nest building score. To nullify any impact of epigenetic/ hormonal modulation on new bone formation, only male rats are being considered in this study.

## Materials and methods

### Ethics statement

All the surgical procedures and other animal experiments were approved by the Institutional Animal Ethics Committee (ILS/IAEC-202-AH/DEC20) of Institute of Life Sciences, Bhubaneswar, India. All our animal experiments were performed in accordance with CPCSEA (Committee for the Purpose of Control and Supervision of Experiments on Animals) guidelines and regulations. We have followed the recommendations provided in the ARRIVE guidelines while reporting the animal study.

### Animals

In this study we have used male Copenhagen rats (4–5 months old, 200–250 g body weight), an inbred strain as a model to study the effect of biomaterial construct on calvarial bone regeneration. Both mice and rats continue to regenerate their skeleton throughout their lifetime. However, due to their large body size and surface area, rats are more favourable than mice in creating critical size surgical defects and bone graft implantations. Moreover owing to their longer life span, bone regeneration patterns can easily be observed for a prolonged time point in rats^23^.

### Housing environment

Male Copenhagen rats were housed in compatible groups in individually ventilated cages (Citizen Industries, Ahmedabad, India) with corncob and wood-shaving bedding (Sparconn Life Sciences, Bengaluru and Kansara scientific Pvt. Ltd., Himachal Pradesh), wood shredding nesting material (Kansara scientific Pvt. Ltd., Himachal Pradesh) and fed with standard laboratory rodent diet (VRK Nutritional Solution, India) and water ad libitum. The animal room was provided with a heating, ventilation, and air-conditioning (HVAC) system. The temperature was maintained at 22 ± 2 °C and relative humidity at 50–70%. The room air change per hour was 16–20. The rats were maintained at 12 h light: 12 h dark cycle in a noise-free environment. The rats were handled and used for experiments as per the Committee for the Purpose of Control and Supervision of Experiments on Animals (CPCSEA) guidelines. The rats were free of viral, bacterial and parasitic pathogens such as Kilham rat virus, Mycoplasma pulmonis, Pneumonia virus of mice, Rat corona virus, REO-3 virus, Rat parvo virus, Sendai, Theiler's murine encephalomyelitis virus, Pin worm (Syphasia Obvelata), ecto-parasite (Polyplax spinulosa).

### Nesting material

All rats were habituated with sterilized wood shredding nesting material (Kansara scientific Pvt. Ltd., Himachal Pradesh) along with nest-let (Ancare) 3 days before surgery. On the day of surgery, the rats were provided with 8 g of fresh wood shredding nesting material and one square piece of nest-let.

### Study design

To measure post osteotomy efficacy of analgesic treatment by tramadol, rats were divided into four groups; treatment group with intraperitoneal injection (T^I/P^), treatment group with oral administration in drinking water (T^Oral^), pre-emptive analgesia control (AnC), and control without any pre-emptive analgesia (Cnt). So, a total of 36 male rats 4–5 months old were used from the in-house colony of Institute of Life Sciences for craniotomy surgery and divided into three groups. Randomly, 12 animals were kept each in the T^Oral^, T^I/P^ group and 6 animals each in the AnC and Cnt. group. The animals were housed for 7 days of acclimatization prior to the surgery. On the day of surgery (0 h), all the rats except for Cnt group were given tramadol at the rate of 10 mg/kg via I/P route as pre-emptive analgesia. From the next day onwards the treatment groups were divided according to the route of administration (I/P and Oral) till 96 h. For better understanding, the group design has been displayed as follows.

**Table:** 

Group		Pre-operative analgesia	Post-operative analgesia
Treatment	T^I/P^	Yes	Yes
	T^Oral^	Yes	Yes
Control	AnC	Yes	No
	Cnt	No	No

### Critical size cranial defect

#### Pre-operative procedures

All the animals were weighed before starting the surgery. Animals were given inhalation anaesthesia with sevoflurane (3–5%) for induction followed by ketamine (80 mg/kg body weight) and xylazine (8 mg/kg body weight) via intraperitoneal injection. Tramadol was also given intraperitoneally (10 mg/kg) to both the experimental animal groups before surgery and then transferred to a sterile bench for shaving. Using a shaving blade hair was removed from the region in between the eyes to the posterior end of the skull. The shaved region was disinfected by alternate swabbing with cotton dipped in povidone-iodine solution followed by 70% alcohol swabs, avoiding contact with the eyes. To prevent dryness of the eye during surgery, lubricating eye drop was applied. Then the animals were transferred to a stage covered with sterile drapes. Using a keyhole drape the body was covered completely and only the site of surgical operation was exposed. The surgical site was again disinfected with a swab of povidone-iodine solution followed by 70% alcohol swabs.

#### Operative procedures

After confirming the achievement of surgical plane of anesthesia (Inhalation of Sevoflurane: 3–5%, I/P injection of Ketamine: 80 mg/kg and Xylazine: 8 mg/kg), a deep longitudinal incision of about 1.5 cm long was made using sterile scissors, scalpel and forceps starting from the area just behind the eyes down to the mid-sagittal area of the skull. The periosteum covering the skull was divided using a sharp scalpel to expose the skull. The skin above the skull was retracted laterally and the calvarium was scored using an 8 mm trephine bur. During the surgery, the skull site was continuously irrigated with sterile normal saline to maintain temperature equilibrium that was generated by the use of trephine burs and to remove the bone chips. With continuous slow speed and gentle pressure with the trephine bur, the critical size calvarial defect was created successfully. After completing the defect, the surgical site was flooded with an ample amount of sterile normal saline. The skin incision was closed with non-absorbable nylon sutures (3-0). The animals were administered with 2.5 ml of normal saline to aid in surgical dehydration and blood loss. Povidone-iodine was applied on the skin at the site of surgery and the animals were wrapped with sterile tissue rolls to provide additional warmth to combat hypothermia of anaesthesia and transferred to IVC cages. Animals were housed singly for 5 days to avoid mutilation at the surgical site.

#### Post-operative procedures

As mentioned in pre-operative procedures, analgesic tramadol hydrochloride was administered to all the animals (10 mg/kg, I/P) 10 to 15 min before surgery, followed by tramadol in drinking water (10 mg/kg) to T^Oral^ group and intraperitoneal injection of tramadol to T^I/P^ group at 12 h interval (twice daily till 96 h). The injectable tramadol hydrochloride was diluted with 0.9% NaCl to achieve a concentration of 2.5 mg/ml so that the injection volume was 0.4 ml per 100 g body weight. For T^Oral^ group, tramadol oral tablets were crushed and mixed with drinking water to prepare a solution having a final concentration of 0.125 mg/ml. To increase the palatability of drinking water, 3% sucrose was added along with the crushed tramadol pills. The other two groups received only sucrose containing water. All the animals were habituated with sucrose water 3 days prior to surgery. The water intake in rats is 8–11% of the total body weight^24^. No analgesia was provided to the AnC group post-surgery.

### Pain assessment tests

To track the metabolic activity and overall health standards of the animals, all the non-invasive methods have been used to study pain management post-surgery.

#### Clinical assessment

Upon observation, no clinical complications like the opening of the wound, skin irritation, or distressed behaviour like guarded behaviour, ill-groomed appearance, hunched posture, and soiled fur were observed.

#### Feed and body weight

Each animal was given with 200 g pelleted feed at the start of experiment i.e., on the day of surgery. The body weights of all the rats were recorded before surgery. The fluctuations in body weight and feed weight were recorded every day till 96 h with the help of a digital weighing balance. To obtain the change in body weight on a particular time point, the initial body weight (before surgery) of an animal was subtracted from its weight on each day. Similarly, for the food intake, the food weight on each day was subtracted from the initial food weight (200 g).

#### Daily water intake

200 ml sucrose containing water with or without tramadol was given to all the rats right after the surgery. The daily water uptake was observed during the early hours of the day starting after 24 h from the day of surgery. To get the intake value, the remaining volume of water was subtracted from the initial volume (200 ml).

#### Grimace scale score

The rat's facial features were photographed each day during the early hours of the day at 8AM to 9 AM without disturbing them. Depending upon the features, the rats of two groups were scored as per the previous scoring method^25^. Orbital tightening, nose/ cheek flattening, ear folding and whisker position were taken as the parameters to score the animals from no pain or normal (0) to obvious pain (2) (Table 1).

**Table 1:** Rat grimace scale scoring scheme for assessment of pain by observing the four action units.

Action units	Baseline	Moderate	Obvious/severe
Orbital tightening	0	1	2
Nose/Cheek flattening	0	1	2
Ear folding	0	1	2
Whisker change	0	1	2

Orbital tightening- Rats in pain exhibit narrowing or squeezing of eyes. The scoring is done accordingly from 0, 1, and 2 if the eyes show normal, slight closure or more closure respectively.

Nose/Cheek flattening- Rats in pain show less bulging of nose and cheek and a flattened appearance of cheek and whisker pad with absence of the crease. The scoring is done accordingly from 0 to 2 if the rat exhibit bulging of cheek, slight flattening or complete flattening respectively.

Ear changes- Rats in pain show ear changes, i.e., the ear turns forwards and outwards forming a pointed shape. The space between the ears also become wider.

Whisker change- As a manifestation of pain sensation in rats, the whisker move forward away from baseline position and bunch together.

Here we have used scoring pattern as follows:

Action unit not present = 0.

Action unit moderately visible = 1.

Action unit pronounced = 2.

#### Nest building score

After surgery, 8 g of fresh wood shredding nesting material was kept inside each cage and the nest-building activity of the rats was photographed each day. Scoring was done as per the previously described method^26^, by considering six parameters scored from 0 to 5 (Table 2).

**Table 2:** Scale for composite nest scoring.

Description	Score
Untouched nest	0
Only manipulation but no nest building	1
Flat nest with a particular shape	2
Cup shaped nest	3
Incomplete dome shaped	4
Complete dome shaped	5

#### Nest-let integration

At the beginning, 2.5 g of a square piece of nest-let was weighed and kept at the top left corner of each cage. The rats actively shred and integrate the nest-let to their nesting material for building a cohesive nest. The pattern of integration was observed and photographed and the weight of the un-shredded nest-let was taken each day till 96 h. To find the weight of the integrated nest-let, the remaining weight was subtracted from 2.5 g (initial weight). For scoring nest-let integration, the overall percentage was calculated as per the below-given formula and was scored from 0 to 5 as per the percentage of integration (Table 3).

$$Percent\;degree\;of\;nestlet\;integration \left( {DOI} \right) = \frac{Initial\;weight\;of\;nestlet - weight\;of\;unshredded\;nestlet }{{Initial\;weight\;of\;nestlet}} \times 100$$

**Table 3:** Scale for nest-let integration scoring.

Description	Score
Untouched	0
Manipulation	1
25% integration	2
50% integration	3
75% integration	4
100% integration	5

Nest building behaviour and grimace scale signify the well-being and activeness of the animal which is an instinct. All the photographs were taken in the early light cycle between 8 and 9 AM. At first, the photographs of the cage along with the animals were taken without disturbing the rats, and then the pictures of the disintegrated nest-let were taken which was followed by weighing of the feed and remaining nest-let. The animal body weight was taken at the last to avoid any unnecessary stress.

### Statistics

GraphPad PRISM Version 8 was used for making the graphs and data analysis. One-way and two-way ANOVA with Tukey’s and Sidak multiple comparison tests were used for statistics. All data were represented as mean ± standard error of mean (SEM). *P* < 0.05 has been taken as a significant value.

## Results

**Figure 1:**
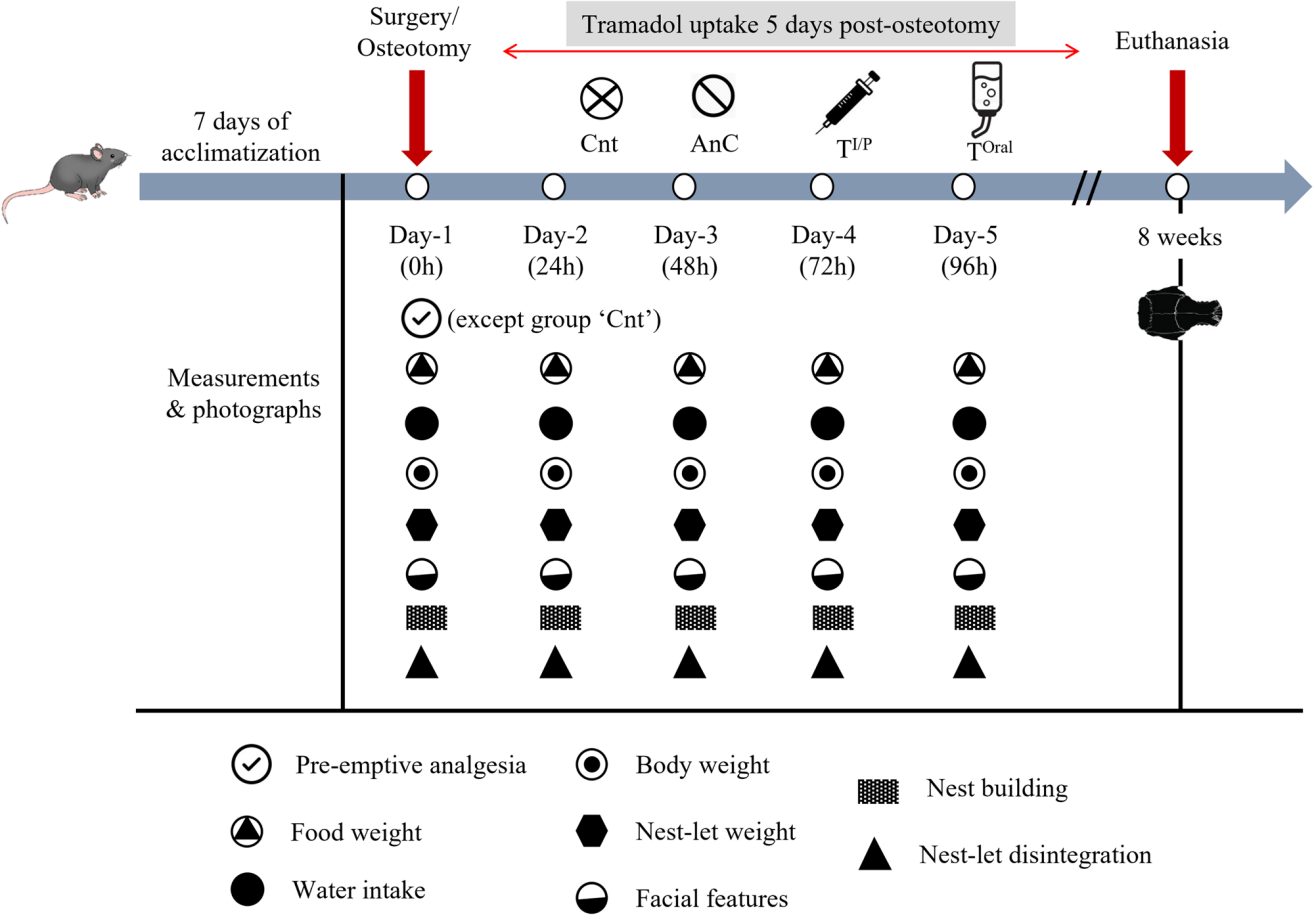
Schematic representation of the timeline of the events and tests performed in the four groups i.e., Cnt, AnC, T^I/P^ and T^Oral^. The day of surgery is considered as starting day of experiment (day-1) when pre-emptive analgesia was injected to the rats. All the parameters were being carefully noted till day 5. All the animals were kept till 8 weeks after which they were euthanized using inhalation anaesthesia (sevoflurane) followed by CO_2_ inhalation for further bone regeneration related studies.

The above-described study allows performing a comparative analysis of the pain management potential between oral and intraperitoneal administration of analgesics post craniotomy surgery by using various non-invasive methods. Observation of rats revealed no physical complications from the surgical process performed and none of the rats manipulated or mutilated their incision sites. No animal died during or post-surgery in this particular study. From the day of surgery (0 h), till 96 h the animals were kept under observation to assess the impact of our study on pain management. All the events and pain assessment measures have been represented schematically in Fig. 1.

### Body weight, food and water intake

**Figure 2:**
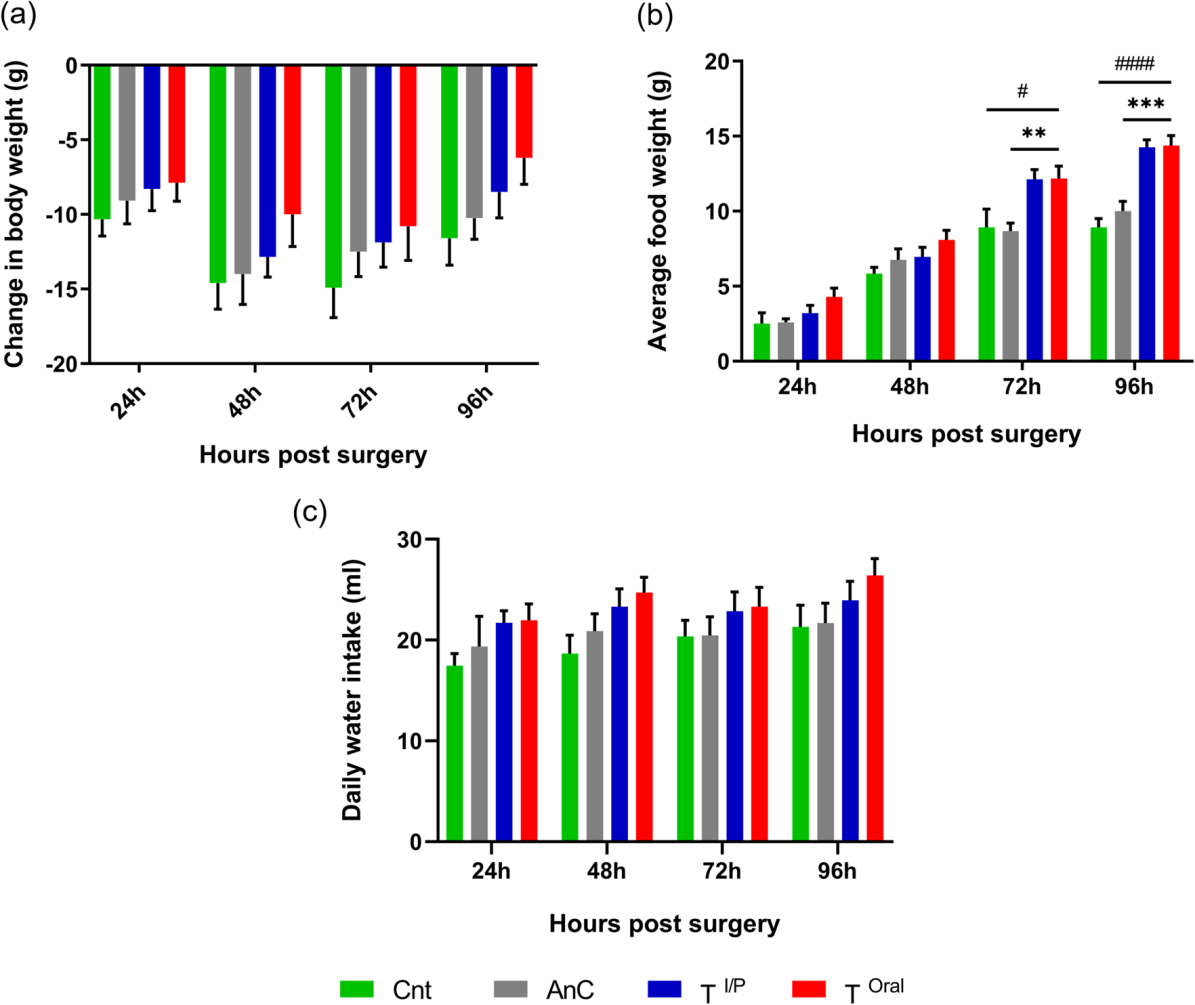
Change in body weight, feed intake and daily water intake. (**a**) Comparative graph representing the gradual reduction in body weight of rats till 48 h followed by slight recovery at 72 and 96 h. (**b**) Graph showing the increasing average food intake per day with significant difference between Cnt, AnC, T^I/P^ and T^Oral^ groups at 72 and 96 h. (**c**) Graph indicating the non-significant difference in daily consumption of water between all the groups. Data are shown as Mean ± SEM. #, *p* < 0.05, **, *p* < 0.001, ***, *p* < 0.0005, ####, *p* < 0.0001. # in comparison with Cnt, * in comparison with AnC.

Body weight and daily food and water intake are the parameters of general well-being and activity. Any fluctuations in these indicate the concerns to reconsider the health of the animals. In our study, after the calvarial surgery, the change in body weight, food weight and water intake were tracked every day till 96 h and was normalized to their respective initial values. Till 48 h, the rats of all groups lost body weight very rapidly irrespective of the control (Cnt and AnC) or analgesic treatment (T^I/P^ and T^Oral^). However, from 72 h onwards gradual recovery was observed in all the groups. Nevertheless, at any of the given time points, no significant difference was observed between the two treatment groups and the control group (Fig. 2a).

The average food intake per day was seen to be increasing with each day in all the groups. At 72 and 96 h, both the treatment groups (T^I/P^ and T^Oral^) showed a significant increase in food intake as compared to both the control groups Cnt and AnC group (At 72 h, Cnt: T^I/P^ and T^Oral^ *p* < 0.05; AnC: T^I/P^ and T^Oral^ *p* < 0.0001. At 96 h, Cnt: T^I/P^ and T^Oral^ *p* < 0.0001, AnC: T^I/P^ and T^Oral^ *p* < 0.0005). However, no significant difference in food intake was observed between the treatment groups (Fig. 2b).

The daily water consumption was found to be comparable at all the time points in all the groups showing no significant difference in the values (Fig. 2c).

From our study, we observed that in all the groups the body weight initially reduced and then started to recover gradually. But the food intake per day was increased with each day having a significant increase from the control group but no significant difference between the treatment groups. Additionally, we found no differential water consumption between the groups.

### Composite pain score/grimace scale

**Figure 3:**
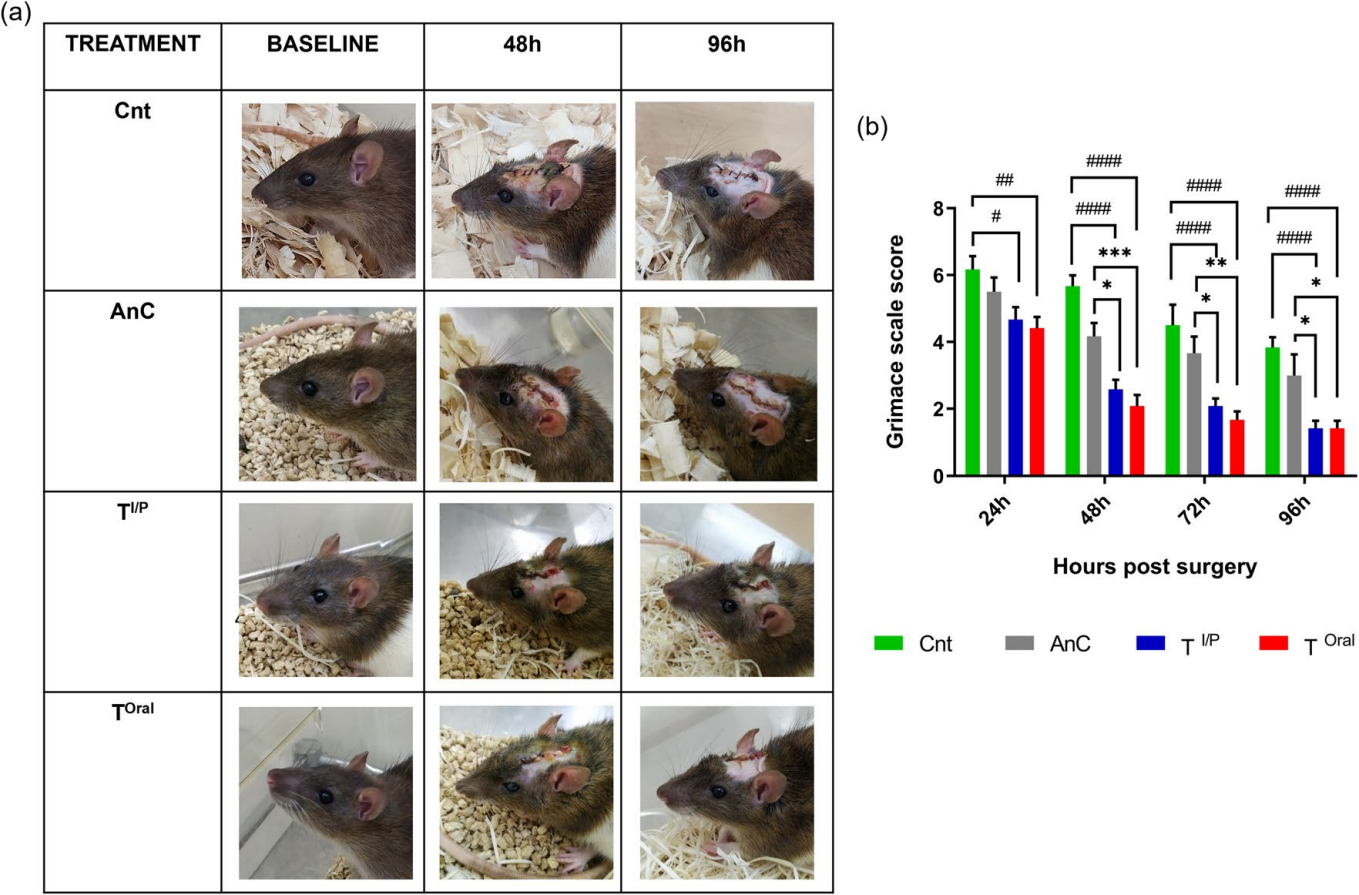
Composite grimace scale scoring. (**a**) Representative photographs of comparative facial features between baseline (before surgery) and treatment groups on day 3 and day 5 post-surgery. (**b**) Graph representing grimace scale scoring between Cnt, AnC, T^I/P^ and T^Oral^ groups at 24, 48, 72 and 96 h post-surgery. Data are shown as Mean ± SEM, # *, *p* < 0.05, ## **, *p* < 0.001, ### ***, *p* < 0.0005, #### ****, *p* < 0.0001. # in comparison with Cnt, * in comparison with AnC.

As animals exhibit similar facial expressions to pain and other emotional states just like humans, in our study rat grimace scale scoring was prepared for pain assessment as described in the methodology. The four action units of the rat grimace scale were observed every day till 96 h. All the action units were assigned the numbers from 0 to 2 to score from no pain to severe pain (Table 1). For a better understanding of the scoring system according to the facial features, between AnC, T^I/P^ and T^Oral^ groups, on 24, 48, 72 and 96 h, a table is presented using some of the representative photographs (Fig. 3a). After assigning the appropriate scores for all the action units of each rat, those were added up to get a final score and the values were represented graphically. We found that within the treatment groups, there was no significant difference in the rat grimace score at any of the time points. However, a significant reduction in grimace scale scoring was observed in both the treatment groups as compared to the control group Cnt at 24 h (T^I/P^, *p* < 0.05, T^Oral^, *p* < 0.001). After 48 h, significant reduction in the score was found in comparison to both the treatment groups (Cnt: T^I/P^, *p* < 0.0001, T^Oral^, *p* < 0.0001; AnC: T^I/P^, *p* < 0.05, T^Oral^, *p* < 0.0005), 72 h (Cnt: T^I/P^, *p* < 0.0001, T^Oral^, *p* < 0.0001; AnC: T^I/P^, *p* < 0.05, T^Oral^, *p* < 0.001) and 96 h (Cnt: T^I/P^, *p* < 0.0001, T^Oral^, *p* < 0.0001; AnC: T^I/P^, *p* < 0.05, T^Oral^, *p* < 0.05) post-surgery (Fig. 3b).

### Nest building and nest-let integration score

As the measure of physical fitness and well-being of animals, the nest-building behaviour and nest-let integration score has been used in this study for pain assessment. In our study, we observed that some of the rats were active in constructing a cohesive nest, whereas others simply constructed temporary sleeping spots with the provided nesting material.

**Figure 4:**
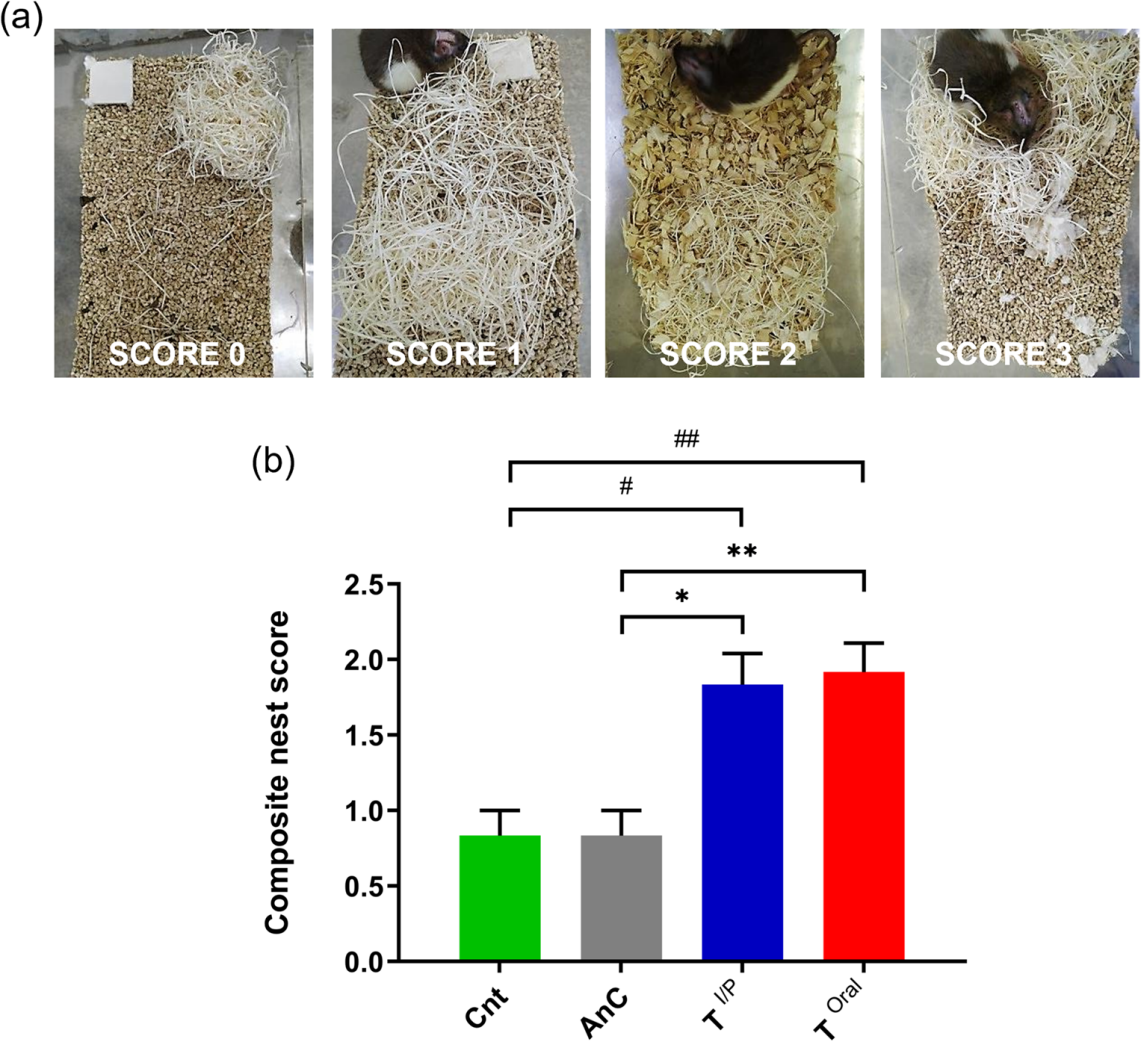
Composite nest building scoring at day 5 post-surgery. (**a**) Representative images of nest building by rats along with the respective scores. (**b**) Graph showing significant difference in composite nest scores between Cnt, AnC and the treatment groups T^I/P^ and T^Oral^ (T^I/P^, *p* < 0.05, T^Oral^, *p* < 0.001), whereas within the treatment groups there is no significant variation of nest score. Data are shown as Mean ± SEM. # *, *p* < 0.05, ## **, *p* < 0.001, # in comparison with Cnt, * in comparison with AnC.

To rate the nest-building ability of the rats, a six-point scoring system was adapted as described in methodology. Starting from a score of 0 signifying untouched nest, to a score of 5 signifying complete dome-shaped nest was assigned (Table 2). Based on visual observation, the nest building scoring was given to the rats (Fig. 4a) which showed a non-significant difference between the two treatment groups, whereas they were found to be more active as compared to both the control groups (T^I/P^, *p* < 0.05, T^Oral^, *p* < 0.001) (Fig. 4b).

**Figure 5:**
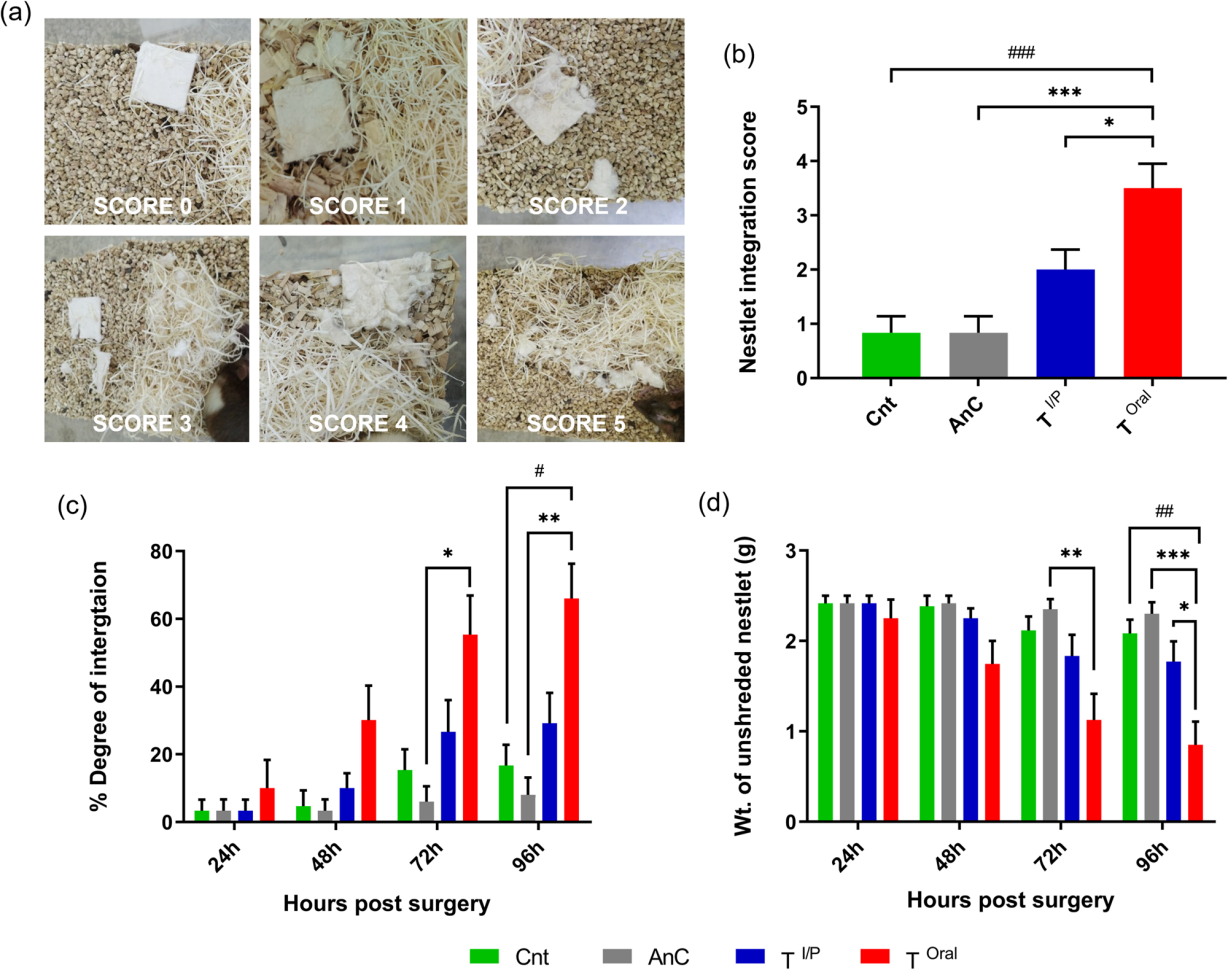
Scoring of nest-let integration to main nest. (**a**) Representative photographs of the shredded nest-lets along with the assigned scores. (**b**) Graph showing the comparison of the nest-let integration scores between the three groups at 96 h. Comparative graphical analysis showing the (**c**) percentage of degree of integration (DOI) and (**d**) the average weight of remaining un-shredded nest-let between the three groups. Data are shown as Mean ± SEM, # *, *p* < 0.05, ## **, *p* < 0.001, ### ***, *p* < 0.0005. # in comparison with Cnt, * in comparison with AnC.

In the nest-let integration study, the percent degree of nest-let integration (DOI) was calculated using the formula described previously. Then the percentage was scored from 0 to 5 starting from untouched nest-let to 100% integration (Table 3). To understand the scoring system, a group of representative photographs of nest-lets after shredding have been scored and given in Fig. 5a.

After 96 h, the percent DOI was calculated for each rat of all the three groups and was scored as per our scoring system. Figure 5b is the graphical representation of the scored data which shows that the T^Oral^ group has a significantly higher integration score as compared to the T^I/P^ group (*p* < 0.05) indicative of their better physical fitness and well-being to build the nest. The percent DOI of all the groups from 24 to 96 h is also represented in Fig. 5c. At 72 h, T^Oral^ was found to have significantly higher DOI as compared to AnC (*p* < 0.05). At 96 h also, the T^Oral^ group demonstrated significant differences in the percent DOI as compared to both the control groups (Cnt: *p* < 0.05, AnC: *p* < 0.001). The weight of the un-shredded nest-let of the three groups has also been portrayed graphically in Fig. 5d, which represents significantly less un-shredded nest-let (*p* < 0.01) in the T^Oral^ group as compared to the T^I/P^ group at 96 h. However, both the groups show significant differences in un-shredded nest-let as compared to the control group AnC both at 72 h (*p* < 0.001) and 96 h (*p* < 0.0005). As compared to the Cnt group, T^Oral^ group showed less amount of un-shredded nest-let at 96 h (*p* < 0.001).

## Discussion

We hypothesized that for post-operative pain management, self-administration (10 mg/kg) of tramadol through drinking water or an intraperitoneal route (10 mg/kg) over a period of 96 h would achieve effective analgesia in Copenhagen rats undergone for craniotomy. Our study aimed to compare both methods of administration. Oral administration of analgesia was aimed to achieve a reduction in pain without any negative impact of restraint stress on the rats. This method is more welfare-friendly because the stress involved in the repeated physical restraining of the animal for drug administration affects hormonal profile, temperature, heart rate and different blood parameters, which may increase the stress, anxiety, risk of post-surgical infection, hemorrhage, and delayed wound healing.

Tramadol is a potent analgesic for mild to moderate pain^27^. It is an agonist of µ-opioid receptor and centrally acting analgesic having structural similarity to codeine and morphine^28^. It has been observed in mice, that continuous postoperative analgesia would be achieved by administrating the drug tramadol through drinking water^29^. In case of rats, voluntary ingestion of tramadol through oral route was found to be an effective analgesic agent^30^.

In our study, we operated on male rats for craniotomy to achieve bone defect which inflicted moderate pain. All rats in four different groups showed the effect of anaesthesia, pain, and analgesia. The study was conducted to observe the comparative effect of analgesia of tramadol by two different routes. We kept two control groups Cnt (without pre-emptive or post-operative analgesia) and AnC (with pre-emptive analgesia but no post-operative analgesia). AnC group was taken to normalize the effect of pre-operative analgesia in the control as well as both the treatment groups. Cnt group was taken to avoid biasness of the effect of pre-emptive analgesia upon the effect of analgesic treatment post-operatively. Interestingly, the rats of the group T^Oral^ showed comparable or better pain management efficacy than the T^I/P^ group and significantly higher pain management effects than both the control groups. To avoid the dehydration due to fluid loss, we administered normal saline immediately after surgery. The animals from all groups started drinking water willingly on their own after recovery from anesthesia and no animal showed any sign of dehydration. However, we did not observe any significant difference between the groups for water consumption. To ensure effective administration of the drug through drinking water, the water was sweetened with sucrose^31^. We used just 3% sucrose solution for a period of 5 days only for the purpose of enhanced palatability for drug administration. Generally, to induce hyperglycemia in rats, palm oil (10%) and sucrose (10%) are used in drinking water for a longer period of 15–16 weeks^32^. Although, we have not checked the glucose concentration in blood, but we assume that such a low concentration of sucrose for a short period of time will not be sufficient enough to induce hyperglycemia.

The reduction in body weight and feed intake after surgery was observed in all four groups till 48 h. This was indicative of pain due to the surgical procedure^33^. This effect was obvious after osteotomy, fluid loss during surgery, hypothermic effect and central nervous system depressive effect of anaesthesia^34^. However, from 72 h onwards all the animals showed gradual recovery with regard to body weight but no significant difference was observed between the two treatment groups and both the control groups (Fig. 2A). The average feed intake was also gradually increased from 72 h onwards in all groups and the analgesic group (T^Oral^ and T^I/P^) showed a significant increase than both the control groups Cnt and AnC (Fig. 2B). The feed and water intake between the two treatment groups was comparable. This effect was suggestive of the potent effect of both types of administration methods for pain alleviation due to craniotomy.

The pain assessment was made by all non-invasive home cage observation methods like rat grimace scale scoring, nest-let integration to nest and nest building score.

A composite pain score or rat grimace scale (RGS) was employed as a non-invasive home cage observation scoring for pain assessment. The grimace scale is proved to be one of the reliable methods in converting facial expressions to score systems among rodents and other species^25^. The pain sensation was obvious due to cranium surgery, so the composite pain score was high at 24 h for all the groups but there was a significant reduction in T^Oral^ and T^I/P^ groups as compared to both Cnt and AnC group at 48, 72 and 96 h (Fig. 3B). The RGS was comparable in both the treatment groups on 48, 72 and 96 h post-surgery indicating both the methods of administration of tramadol were equally effective in post-operative pain management.

Nest-building behaviour of mice has been known as the measure of their physical fitness and well-being^35^. Similarly, a healthy rat can use nesting material for nest building purpose and this behaviour is acquired and learned when nesting material is provided from birth^36^. We included this nest building and nest-let integration score as one of the non-invasive measures of rat’s wellbeing. All the animals undergoing the experimental procedure were well habituated to nesting material. To have a better understanding of the nest-building behaviour we took the nest score of 96 h post-surgery into consideration. The nest scores of both the treatment groups were significantly higher than both the control groups; Cnt and AnC indicating better pain alleviation and increased activity (Fig. 4B) which is reflective of the reduced effect of anaesthesia and reduced pain sensation. However, in this pain management assessment also both the analgesia groups showed a comparable effect.

The time-to-integrate-to-nest test (TINT) is used as an indicator of well-being in laboratory mice^37^. The results of TINT show variation for different painful procedures^38,39^. In this study we have used a modified TINT test for rats. Here instead of looking into the time, we observed the explorative behaviour of rats and also the degree of disintegration of new nest building material (nest-let). Nest-let shredding is an indirect measure to indicate the extent of the natural nest-building ability of rats by shredding any given material^36^. Explorative behaviour in rodents is indicative of well-being, anxiety and activity^40^. Animals in all groups showed reduced exploration and disintegration of nest-let during the first 24 h period post-surgery which may be due to the effect of anaesthesia and pain. But during the period of 96 h of observation, between two treatment groups, the T^Oral^ group showed a higher level of exploration and shredding of the nest-let. Exploration and integration were also there in the T^I/P^ group and it was significantly higher than in the control groups Cnt and AnC (Fig. 5C). This observation indicates that the T^Oral^ treatment has comparable pain management efficacy as that of T^I/P^ and a stronger positive effect on wellbeing than T^I/P^, Cnt and AnC group.

Rodents like mice and rats that are under osteotomy pain show guarding behaviour and an ill-groomed appearance^41^. Though we have not employed any scoring pattern, the rats in neither of the groups did show any guarding behaviour rather they used to groom themselves well. This observation suggests a sufficient reduction of pain sensation.

Our study protocol included only male Copenhagen rats as it is a sub-study of a major experiment. We have not considered the female counterpart of the strain, which would have exhibited similar or different outcomes. In addition to our study, the strain-specific conclusion can be drawn after considering the female rat pain behaviour study. Nevertheless, the post-operative analgesic management could be further refined with the use of sweetened flavored jelly-based oral medication or injection and oral combination therapy. Pain assessment studies can be done with different concentrations of analgesics for better understanding. Oral analgesia which has added welfare effects may be employed for other biological procedures with severe pain implications.

## Conclusion

In this study we found that both the treatment groups showed significant differences in pain management as compared to both the control groups. However, any significant difference was not found between the treatment groups for all parameters except the nest-let integration score and shredding rate of the nest-let. Overall, our study outcomes suggest that both oral and intraperitoneal route of administration of analgesia have comparable effect towards postoperative pain management. However, oral/self-administration methods have the additional benefit of imparting continuous and stress-free pain management in the rat craniotomy model. We believe that our study can be helpful for future researchers in refining the post-operative pain management strategies while performing painful surgical procedures.

## Data Availability

The datasets generated and/or analyzed during the current study are available from the corresponding author on reasonable request.
